# Evaluation of the impact of reliance on the regulatory performance in the South African Health Products Regulatory Authority: implications for African regulatory authorities

**DOI:** 10.3389/fmed.2023.1265058

**Published:** 2023-10-23

**Authors:** Lorraine Danks, Boitumelo Semete-Makokotlela, Kennedy Otwombe, Yashmika Parag, Stuart Walker, Sam Salek

**Affiliations:** ^1^South African Health Products Regulatory Authority (SAHPRA), Pretoria, South Africa; ^2^Perinatal HIV Research Unit, Division of Epidemiology and Biostatistics, Faculty of Health Sciences, University of Witwatersrand, Johannesburg, South Africa; ^3^Centre for Innovation in Regulatory Science, London, United Kingdom; ^4^School of Life and Medical Sciences, University of Hertfordshire, Hatfield, United Kingdom; ^5^Institute for Medicines Development, London, United Kingdom

**Keywords:** abridged review, backlogs, increased patient access, national regulatory authorities, recognized regulatory authorities, reliance implementation

## Abstract

**Introduction:**

The World Health Organization (WHO) advocates the use of reliance practices to enable national regulatory authorities (NRAs) to improve patients’ access to medicines. This study considered whether reliance review translates into swifter medicine authorization.

**Methods:**

Abridged review outcomes were examined for New Chemical Entity (NCE) and generic applications to the South African Health Products Regulatory Authority (SAHPRA) in Chemistry, Manufacturing and Controls (CMC) and clinical/bioequivalence (BE), as well as overall NCE authorization times.

**Results:**

SAHPRA NCE CMC review time was 91 days (abridged) vs. 179 days (full), applicant response time was 34 vs. 105 days, respectively, and there was a >2-fold time reduction for abridged vs. full CMC review (125 vs. 284 days). There was a 99-day decrease in clinical approval time through an abridged review (230 vs. 329 days) and a decrease in marketing authorization time for NCE abridged assessment (446 vs. 619 days). SAHPRA review time for generic applications was 97 days (abridged) vs. 191 days (full); applicant response time was 26 days (abridged) vs. 81 days (full) and there was a >2-fold time reduction for CMC and BE abridged vs. full review (122 vs. 272 days).

**Conclusion:**

These results clearly support World Health Organization recommendations for the use of reliance-based regulatory review to expedite the worldwide availability of safe, effective and needed medications.

## 1. Introduction

The World Health Organization (WHO) estimates that there are approximately 2 billion people who are unable to access essential medicines (1). The mandate of every national regulatory authority (NRA) is to ensure patients’ access to quality-assured, safe and effective medicines (2, 3). To assist in strengthening regulatory systems within NRAs of low-to-middle-income countries, the WHO recently published Good Regulatory Practices (GRP) and Good Reliance Practices (GRelP) guidelines (3–5). These high-level documents provide guidance to NRAs in terms of improving their regulation of health products, and further encourages collaboration between NRAs and both regional and international agencies and/or institutions. As many NRAs do not have sufficient capacity regarding expertise or financial resources to fulfill their mandate of ensuring competitively priced medicines for their citizens, the implementation of reliance review practices is critical. This is to avoid duplication of efforts by NRAs and to effectively utilize the available local resources by focusing their regulatory endeavors on country-specific tasks, such as post-marketing surveillance, regulation of local manufacturing and distribution, as well as vigilance activities (3). Facilitated regulatory review pathways play a pivotal role in, not only conserving regulatory effort and focusing on the broader regulatory tasks assigned to an NRA, but also in the expeditious authorization of medicines.

The WHO defines reliance as “the act whereby the regulatory authority in one jurisdiction takes into account and gives significant weight to assessments performed by another regulatory authority or trusted institution, or to any other authoritative information, in reaching its own decision. The relying authority remains independent, responsible and accountable for the decisions taken, even when it relies on the decisions, assessments and information of others (3)”. The WHO further explains abridged regulatory pathways, wherein an abridged assessment of the quality, safety and efficacy data is carried out by an NRA through reliance on prior work by a reference agency by utilizing the latter’s assessment reports. In these instances, only the country-specific requirements, for example, the stability of the product given the country’s climatic zone, are reviewed by an NRA with the relying regulator retaining sovereignty of decision making (3). Verification of sameness underpins this practice, namely that the product submitted to the relying NRA is in all essential aspects identical to the product that was authorized by the reference regulatory agency. The GRelP flows out of the GRP principles, and is characterized by its universality, in that it may be applied by any NRAs, irrespective of their maturity level or resources (3).

According to Section 27 of the South African Constitution, every individual in South Africa is entitled to have access to healthcare as a basic human right (6). This includes access to generic medicines, which, as these are as a rule less expensive than the innovator product (7), is an essential element in fulfilling the basic tenets of human rights. Unfortunately, medicine application backlogs in regulatory agencies are not uncommon (8–11), and these result in restricted access of affordable medicines.

When the South African Health Products Regulatory Authority (SAHPRA) was established in February 2018, it inherited a backlog of approximately 16,000 marketing authorization and variation applications from its predecessor, the Medicines Control Council (MCC) (12). The backlog was created over a number of years prior to the establishment of SAHPRA due to limited resource capacity, diminished financial capabilities, as well as lack of availability of an electronic tracking system. Between 2011 and 2017 it took the MCC a median of 2,092 calendar days to register a new product application (13). The MCC conducted full reviews of all aspects of quality, safety and efficacy for all applications received and, in 2017, the overall regulatory median approval time for marketing authorization of an application was 1,141 calendar days. It was noted that this was a 14% decrease in time compared to 2016, despite an increase of 27% in marketing authorization applications received (14). Despite this improvement in review turnaround time, there was still an ever-growing backlog of applications as the time it took SAHPRA to authorize new products was still too long to promote patients’ access to essential medicines.

To expeditiously deal with inherited applications, SAHPRA set up a dedicated Backlog Clearance Project (BCP), with specific therapeutic resubmission windows (RWs), through internal and external funding and with expertise to clear the application backlog. One of the pivotal regulatory strategies in dealing with this backlog was the implementation of a reliance pathway. In addition, SAHPRA, as part of their long-term planning, optimized their review processes and included risk-based assessment approaches, also addressing the staff shortage and the establishment of robust information and quality management systems (12). This was enabled through a revised legislative framework, namely Section 2B(2) of the Medicines and Related Substances Act No. 101 of 1965 (as amended by Act 72 of 2008 and Act 14 of 2015) that ruled reliance and work-sharing practices permissible (15). By December 2022, the inherited application backlog had been successfully cleared by SAHPRA. This study aims to review the implementation of GRelP within the South African regulatory authority, with specific focus on the abridged review process in the SAHPRA Backlog Clearance Project.

The objectives were to

- compare approval timelines between abridged and full review for New Chemical Entities (NCEs) and generic product applications, stratifying the review times between clinical and Chemistry, Manufacturing & Controls (CMC);
- compare marketing authorization timelines for full and abridged review of NCE applications;
- engage with BCP reviewers regarding the effectiveness of the implementation of abridged review;
- perform an analysis on the similarity of the SAHPRA-authorized NCE labeling vs. that approved by the reference agency, to establish whether alignment was maintained through the SAHPRA BCP abridged clinical review pathway; and
- provide recommendations for process improvement and a strategy for full reliance implementation by SAHPRA.

## 2. Methods

This study provides a comparison between full and abridged review timelines for NCE and generic product applications in the SAHPRA BCP between August 2019 to December 2022. Analyses of CMC and clinical/bioequivalence (BE) review metrics for the two types of applications, assessed according to reliance principles, were performed. Using the entire sampling frame, the relevant abridged review metrics were compared with those of the same types of products of applications or molecules that were assessed via the full review pathway within the same project.

There was no separate analysis of the administrative part of the review process; however, this was taken into account when the overall review timeline was calculated. It should be noted that all applications, regardless of whether these followed the full or abridged review pathway, followed the same administrative process, namely receipt and validation, administrative and technical screening and subsequent allocation to assessors.

### 2.1. Selection of NCE applications

All the NCEs applications received in the BCP were identified. These consisted of applications for molecules where non-clinical and clinical data or literature studies were included as evidence of safety and efficacy, or for products containing previously registered molecules, but in a novel dosage form or novel fixed-dose combination, not previously authorized in South Africa (incrementally innovative products) (16). Furthermore, any active pharmaceutical ingredient (API) not yet registered by SAHPRA was considered a new chemical entity, regardless of whether the molecule had already been registered by other regulatory authorities. These NCEs were spread over multiple therapeutic areas, and included oncology therapies, central nervous system therapies, human immunodeficiency virus and tuberculosis products, multiple sclerosis, attention-deficit/hyperactivity disorder and narcolepsy treatments, anti-infectives/antibiotics, cardiovascular (anti-hypertensive and anticoagulant) and diabetes medicines, and non-steroidal anti-inflammatory drugs and anti-inflammatory agents, as well as hormonal preparations.

### 2.2. Exclusion criteria

- Any responses to MCC recommendations issued to the applicant prior to the Go-Live of the BCP in August 2019, namely if the resubmitted applications contained responses to query letters applicants had received from the MCC; only applications being reviewed for the first time were included in the study cohort.
- Any NCEs that were deemed “low-risk” products and had followed an abbreviated CMC and/or clinical review pathway.
- Any applications where the applicant had withdrawn the application prior to finalization of evaluation.
- Any NCE applications that were clones of products already authorized in South Africa.

Due to the exclusion criteria, only 72 and 77 NCE applications were studied in terms of CMC and clinical review timelines, respectively.

It is important to note that evaluation pathways in SAHPRA may differ for the CMC and clinical assessment of the same application, in that CMC might undergo abridged review, while the clinical data are fully reviewed. This is dependent on the quality and type of the assessment reports supplied, but also widens the use of reliance, by not limiting an application to the same pathway or reference regulatory authority (RRA) for the two sections (17).

The remaining NCE applications were divided into four groups:

1. Applications where the CMC aspects were reviewed in full (*n* = 29).
2. Applications where the CMC aspects followed an abridged review pathway (*n* = 43).
3. Applications where the clinical data were reviewed in full (*n* = 27).
4. Applications where the clinical data followed an abridged review pathway (*n* = 50).

Metrics for three main milestones, namely CMC and clinical assessment and marketing authorization, were collected and collated for each application (Table 1).

**Table 1:** Metrics compiled for the Backlog Clearance Project New Chemical Entity applications.

Start date	Date of official receipt of application (that is date on application letter)
CMC assessment	Date application was first allocated for CMC evaluation
	Period in calendar days application was in CMC review with SAHPRA
	Period in calendar days application was with applicant, responding to CMC queries raised by SAHPRA
	Date of finalization of review of CMC section of application
Clinical assessment	Date application was first allocated for clinical evaluation
	Period in calendar days application was in clinical review with SAHPRA
	Period in calendar days application was with applicant, responding to clinical queries raised by SAHPRA
	Date of finalization of review of clinical section of application
Finalization date	Date of marketing authorization (registration) of application by SAHPRA

CMC, Chemistry, Manufacturing and Controls; SAHPRA, South African Health Products Regulatory Authority.

### 2.3. Selection of generic product applications

The generic product application cohort for this study was compiled by selecting the predominant molecule(s) in each RW in the BCP with a relatively even split between the number of abridged and full review applications. This selection excluded RW 1 (containing mostly NCEs) and RWs 8, 10, 11, and 12, where many applications underwent CMC risk-based assessment. Furthermore, during RW 7, an intervention was instituted to enhance the abridged evaluation process. Therefore, it was decided to include an equal number of molecules from the RWs prior to the intervention (7 molecules), and during/after it (7 molecules), to avoid possible distortion of data due to optimizations in the process from RW 7 onwards (Table 2). Using this sample, an investigation was conducted into the CMC review time for 73 abridged review applications and 123 full review applications in the BCP (*n* = 196); however, exclusion criteria were further applied to the selection.

**Table 2:** Predominant generic molecules selected from the Backlog Clearance Project resubmission windows for the CMC study cohort analyses.

Resubmission window	API	No. of abridged review applications	No. of full review applications	Total applications per API
Resubmission window 2	Docetaxel	4	3	7
	Bortezomib	3	7	10
Resubmission window 3	Olanzapine	6	6	12
	Duloxetine	5	11	16
Resubmission window 4	Moxifloxacin	3	11	14
Resubmission window 5	Metformin	4	8	12
Resubmission window 6	Montelukast	5	10	15
Resubmission window 7	Rosuvastatin	7	6	13
	Ezetimibe	6	6	12
	Telmisartan	5	14	19
Resubmission window 9	Rizatriptan	3	3	6
	Solifenacin	9	8	17
	Pregabalin	7	14	21
	Tadalafil	6	16	22
Total		73	123	196

API, active pharmaceutical ingredient; CMC, Chemistry, Manufacturing and Controls.

### 2.4. Exclusion criteria

- Any responses to MCC recommendations issued to the applicant prior to the initiation of the BCP in August 2019, namely if the resubmitted applications contained responses to query letters applicants had received from the MCC; only applications being reviewed for the first time were included in the study cohort.
- Any generic product applications that were deemed “low-risk” products and had followed a risk-based CMC assessment.
- Any applications where the applicant had withdrawn the application prior to finalization of evaluation.
- Any generic product applications that were clones of products already authorized in South Africa.
- Any line extension(s) of an already authorized product in South Africa.
- Any generic product application where the CMC data were assessed via internal SAHPRA reliance, that is based on in-house SAHPRA assessment reports of another identical product.

After the exclusions, 153 generic product applications remained and were divided into two groups:

1. Applications where the CMC aspects followed an abridged review pathway (*n* = 81).
2. Applications where the CMC aspects were reviewed in full (*n* = 72).

It is worth noting that reference to CMC assessment for generic molecules also includes review of BE data, as the two sections are assessed by the same evaluation unit in SAHPRA, whereas most other regulators review BE studies via the clinical review streams. Moreover, no analyses on different clinical review pathways for generic products were conducted, as SAHPRA follows, as a rule, internal reliance when assessing these products, namely alignment of generic product labeling with the latest SAHPRA-approved innovator’s Professional Information (PI). Metrics for the CMC assessment milestones were collected and collated for each application (Table 3).

**Table 3:** Metrics compiled for the Backlog Clearance Project generic product applications.

CMC assessment	Date application was first allocated for CMC evaluation
	Period in calendar days application was in CMC review with SAHPRA
	Period in calendar days application was with applicant, responding to CMC queries raised by SAHPRA
	Date of finalization of review of CMC section of application

CMC, Chemistry, Manufacturing and Controls; SAHPRA, South African Health Products Regulatory Authority.

### 2.5. Assessors’ questionnaire and focus group discussions

A questionnaire dealing retrospectively with the implementation of the abridged review pathway in SAHPRA BCP was compiled and shared with its CMC and clinical assessors. The survey investigated the perceived advantages and challenges to successful implementation of abridged review in the BCP. It further sought whether the unredacted RRA assessment reports were sufficient for ascertaining the sameness between the RRA and SAHPRA product, thus enabling reliance, and whether consultation of public assessment reports (PARs) contributed to the reliance review of an application. Furthermore, a virtual focus group discussion was held with the CMC reviewers to discuss the outcomes from the questionnaire, which included the following items:

1. What were the main challenges in implementing a reliance (abridged review) strategy in SAHPRA?
2. What did you consider were the main advantages?
3. Were the unredacted assessment reports submitted sufficient to allow for abridged review of the applications?
4. Did you consult any public assessment reports and, if so, did you find this helpful?
5. Any other comments?

### 2.6. Ethics approval

The study was approved by Health, Science, Engineering and Technology ECDA, University of Hertfordshire, United Kingdom [Reference Protocol number: LMS/PGR/UH/05160].

## 3. Results

### 3.1. NCE abridged vs. full CMC review timelines

Unredacted RRA CMC assessment reports were received for 43 of the 72 NCE applications. Most applications (31/43) contained assessment reports from the European Medicines Agency (EMA), with 10 applications containing reports from the Australia Therapeutic Goods Administration (TGA). Reports from Swissmedic and the United Kingdom Medicines and Healthcare products Regulatory Agency (MHRA) were included in only two applications each.

**Figure 1:**
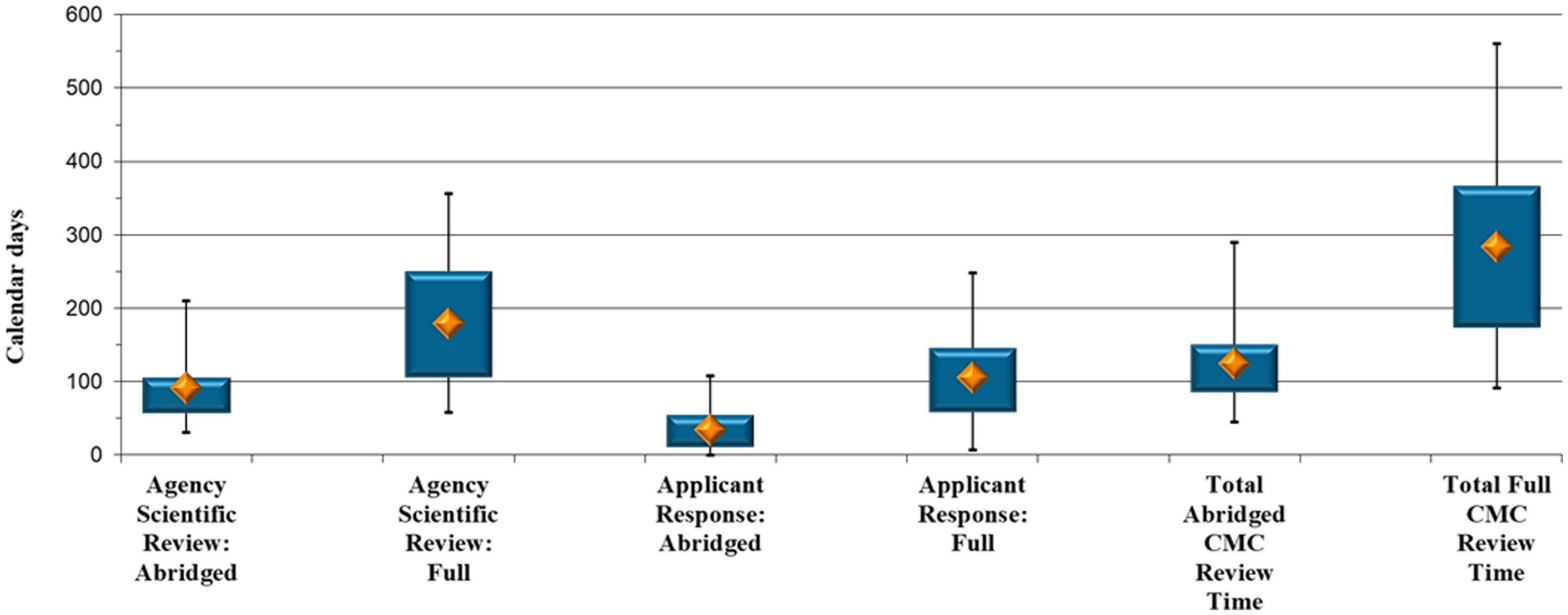
New Chemical Entity comparison: Abridged vs. full Chemistry, Manufacturing and Controls review timelines (calendar days).

Analyses were performed on the 29 NCE applications where the CMC section was reviewed via the full review pathway and the 43 NCE applications that underwent an abridged CMC review. SAHPRA’s scientific review time during the two types of CMC assessment, as well as the time it required applicants to respond to queries raised by SAHPRA after full and abridged CMC review, respectively, were furthermore examined (Figure 1).

SAHPRA took a median of 91 calendar days to finalize an abridged assessment vs. a median of 179 calendar days to perform a full review of an NCE CMC information (*p* < 0.001) (Figure 1). The applications spent double the time with SAHPRA for initial and subsequent response assessment when the CMC aspects were reviewed in full.

When reviewing applicants’ response timeframes (Figure 1), a median of 34 calendar days was necessary for an applicant to respond to CMC queries raised by SAHPRA after an abridged review vs. 105 calendar days to provide a response after a full review (*p* < 0.001). It is evident (Figure 1) that queries arising during an abridged CMC review were responded to by applicants in a significantly shorter timeframe, namely a third of that required for the full review. It is suspected that this was due to the extent or the data requirements of the queries generated when the CMC data were reviewed in full. Moreover, during full review, the closed parts of the Active Pharmaceutical Ingredient Master File (APIMF) are assessed, and when queries are raised pertaining to this section, responses by API manufacturers are required. This leads to increased applicant response time.

Furthermore, the total CMC evaluation time was analyzed, namely the time taken to review the data from the day of allocation for initial assessment until the approval of the CMC section of the specific application. The median duration for completing an abridged CMC assessment of an NCE was 125 calendar days compared with 284 calendar days for a full review (*p* < 0.001) (Figure 1), representing a more than two-fold increase.

### 3.2. NCE abridged vs. full clinical review timelines

Unredacted clinical assessment reports were received for 50 of the 77 NCE applications included in the study cohort. Most applications (36) contained assessment reports from the EMA. As with the unredacted RRA CMC assessment reports, reports from the TGA were the second most prolific, contained in 12 applications. A few reports were also received from Swissmedic (2), the MHRA (2), the US Food and Drug Administration (FDA) (1) and Health Canada (1).

An assessment was conducted on the 27 NCE applications where the clinical data were reviewed through the full review pathway and the 50 NCE applications that underwent an abridged clinical review.

The time spent by SAHPRA reviewing the clinical data, as well as the time it took applicants to respond to questions raised by the agency, was again evaluated. The total clinical evaluation time was also analyzed (from the day of assessment allocation until the approval of the clinical data).

**Figure 2:**
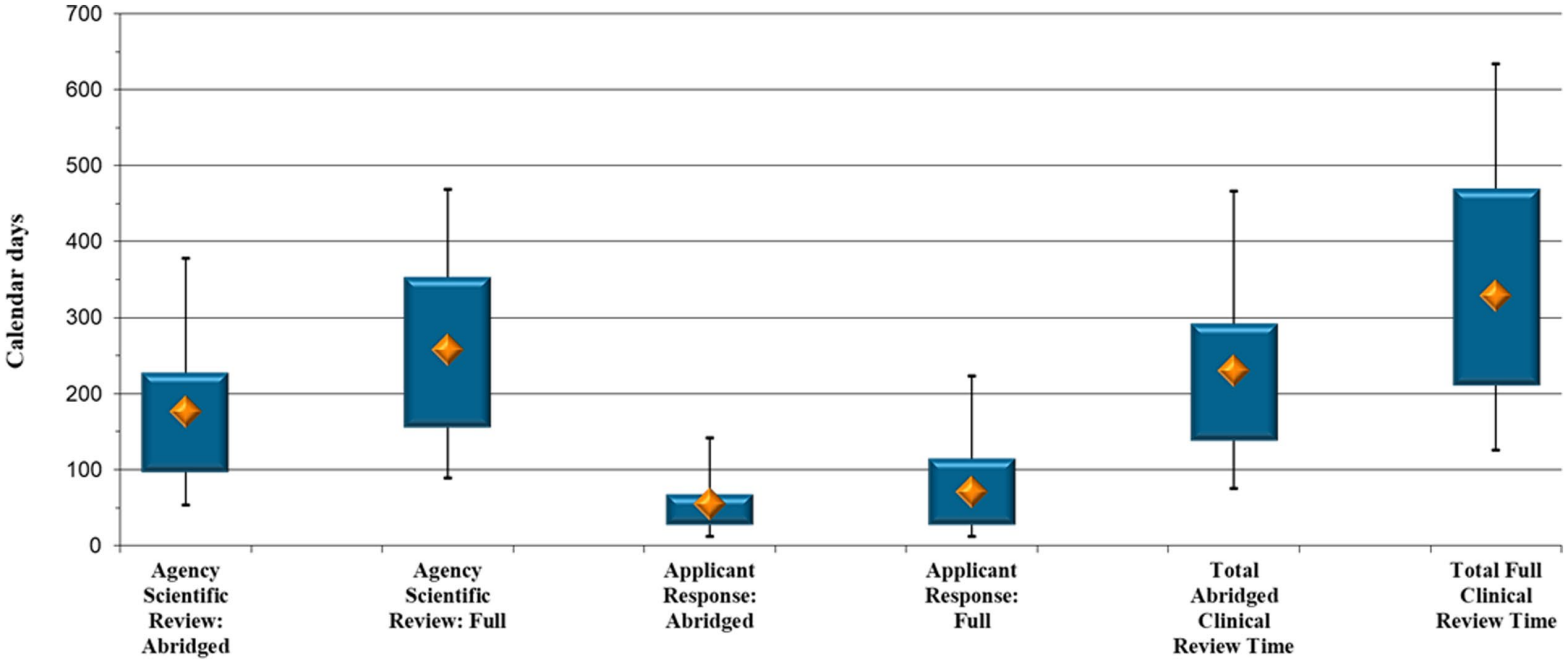
New Chemical Entity comparison: Abridged vs. full clinical review timelines (calendar days).

SAHPRA’s median scientific review time was shorter for clinical data assessed in an abridged manner through reliance (176 calendar days) compared with that via the full review pathway (258 calendar days) (*p* < 0.05) (Figure 2). It was further noted that there was little difference between the time it took applicants to respond to the clinical queries raised by SAHPRA via the abridged and full review pathways, namely 16 additional days for submission of a response to queries raised during full clinical evaluation (55 compared with 71 calendar days, *p* = 0.93).

When comparing the collective time the 50 NCE clinical abridged and the 27 NCE clinical full review applications were under assessment, the results in Figure 2 point to a median of 230 calendar days to complete an abridged review and a median of 329 calendar days to complete a full assessment of NCE clinical data (*p* = 0.027), that is, an additional 99 days.

### 3.3. Alignment of labeling between SAHPRA and reference agencies

To further assess the effectiveness of the implementation of the abridged clinical review, an analysis was conducted on the 50 NCE applications to ascertain the level of alignment between the SAHPRA-approved labeling and that from the relied-upon reference agency. For the majority of NCE applications, the applicants applied for reliance using the EMA Summary of Product Characteristics (SmPC), with the Australian TGA labeling relied on the second most often. Less reliance was placed on labeling from the US FDA, Swissmedic and the UK MHRA.

Certain key sections of the South African Professional Information (SA PI) were compared with the reference agency SmPC, with the following results:

4.1 Therapeutic Indications: 40% non-alignment (20/50).
4.2 Posology and Method of Administration: 44% non-alignment (22/50).
4.3 Contraindications: 74% non-alignment (37/50).
4.4 Special Warnings and Precautions for Use: 60% non-alignment (30/50).
4.5 Fertility, Pregnancy & Lactation: 84% non-alignment (42/50).

There was a high level of non-alignment between the SAHPRA- and RRA-approved labeling, especially in section 4.6 pertaining to fertility, pregnancy and lactation, with 84% of SA PIs not aligning with the SmPCs. A further area of misalignment was that regarding contraindications, with 37 of the 50 SA PIs containing additional contraindications. In one instance, 13 additional contraindications, not reflected in the EMA SmPC, were included in the SA PI. Additional special warnings and precautions were also included in 30 of the SA PIs. Furthermore, the SAHPRA-approved information was more restrictive in nature with deletion of references to “rare” and the removal of information pertaining to benefit-risk determinations by healthcare professionals. Moreover, additional black box warnings were included in some of the SA PIs.

### 3.4. Impact of the NCE reliance review on marketing authorization

A final analysis was carried out to assess the impact of reliance review on the overall authorization time of NCEs. The analysis comprised of two cohorts of NCE applications:

1. where both the CMC and clinical reviews were conducted via an abridged review pathway, and
2. where both the CMC and clinical reviews were performed using the full review pathway.

The marketing authorization time was determined from the day of resubmission of the application in the BCP until authorization thereof by SAHPRA (in calendar days). The results indicated that it took less time to authorize an NCE where both the CMC and clinical reviews were conducted in an abridged manner, that is a median of 446 days (*p* = 0.0005). It took an additional 173 days to register an NCE, when both the CMC and clinical sections were assessed in full (median of 619 days).

### 3.5. Generic molecules—CMC assessment

**Figure 3:**
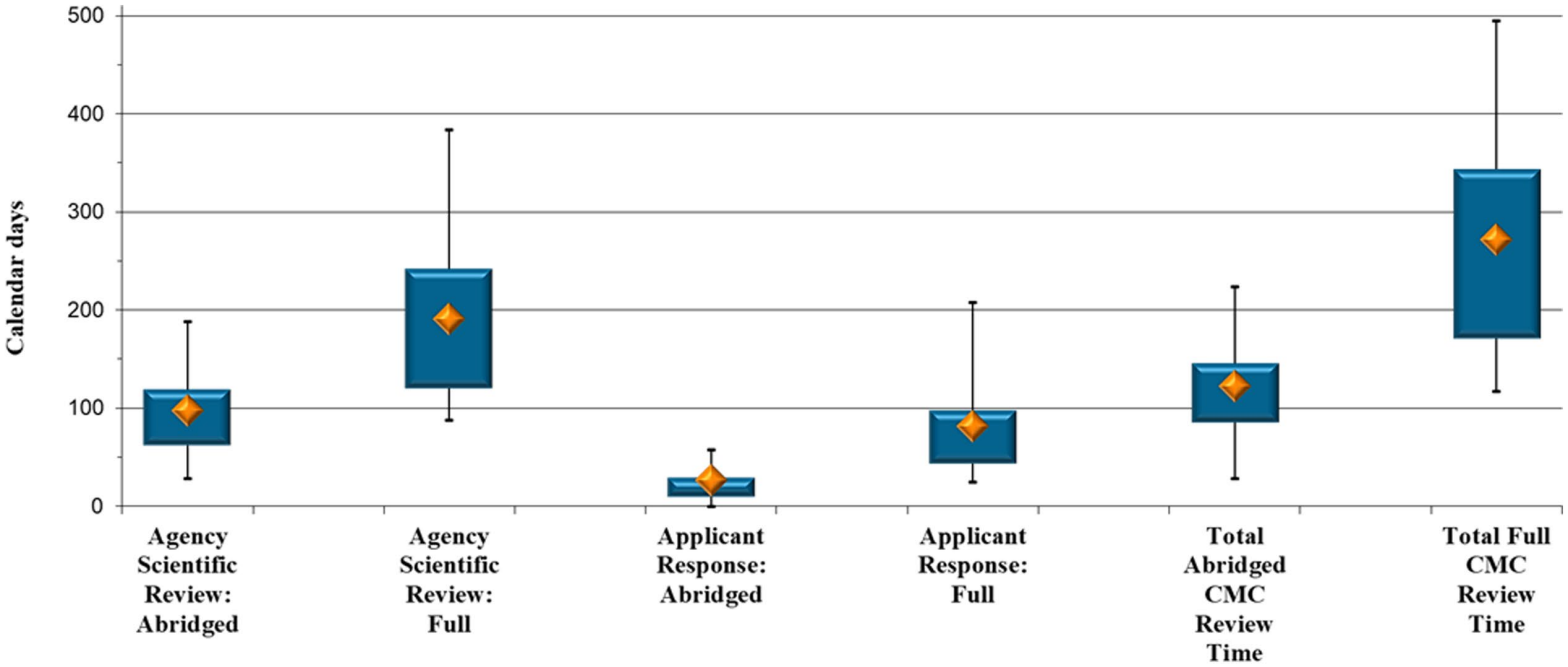
Generic comparison: Abridged vs. full Chemistry, Manufacturing and Controls review timelines (calendar days).

The cohort consisted of a total of 153 generic product applications, divided into 81 applications where the CMC data were assessed via the abridged review pathway and 72 applications that underwent full review of the CMC section (Figure 3).

Once more, analyses were performed to ascertain the time, in terms of each review pathway, the generic product applications were either with SAHPRA or with the applicant (Figure 3). SAHPRA took a median of 97 calendar days to finalize an abridged assessment vs. a median of 191 days to perform a full review of the CMC information (*p* < 0.001); that is, half the time. There was, furthermore, a median of 26 days for an applicant to respond to CMC queries raised by SAHPRA after an abridged review, vs. 81 days to provide a response to queries raised after a full CMC review (*p* < 0.001) (Figure 3). As with NCE data, it takes an applicant approximately three times longer to respond to CMC queries raised after a full review of the data of a generic product. With generic applications, queries pertaining to the BE data further lengthen the applicant response time. The maximum for the full CMC review (496 days) was also considerably higher than that for the abridged review (224 days), as the number and type of queries raised necessitated additional time to compile the requested information, which, in some cases, led to several query rounds (Figure 3).

The total CMC evaluation and approval time is also reflected in Figure 3. The results indicate a median of 122 calendar days to complete an abridged CMC assessment of a generic product, vs. a median of 272 days to complete a full CMC assessment (*p* < 0.001). The results indicate a marked reduction (55%) in overall CMC review time through an abridged review pathway.

### 3.6. Comparison between abridged and full CMC review timelines for NCE and generic product applications

**Figure 4:**
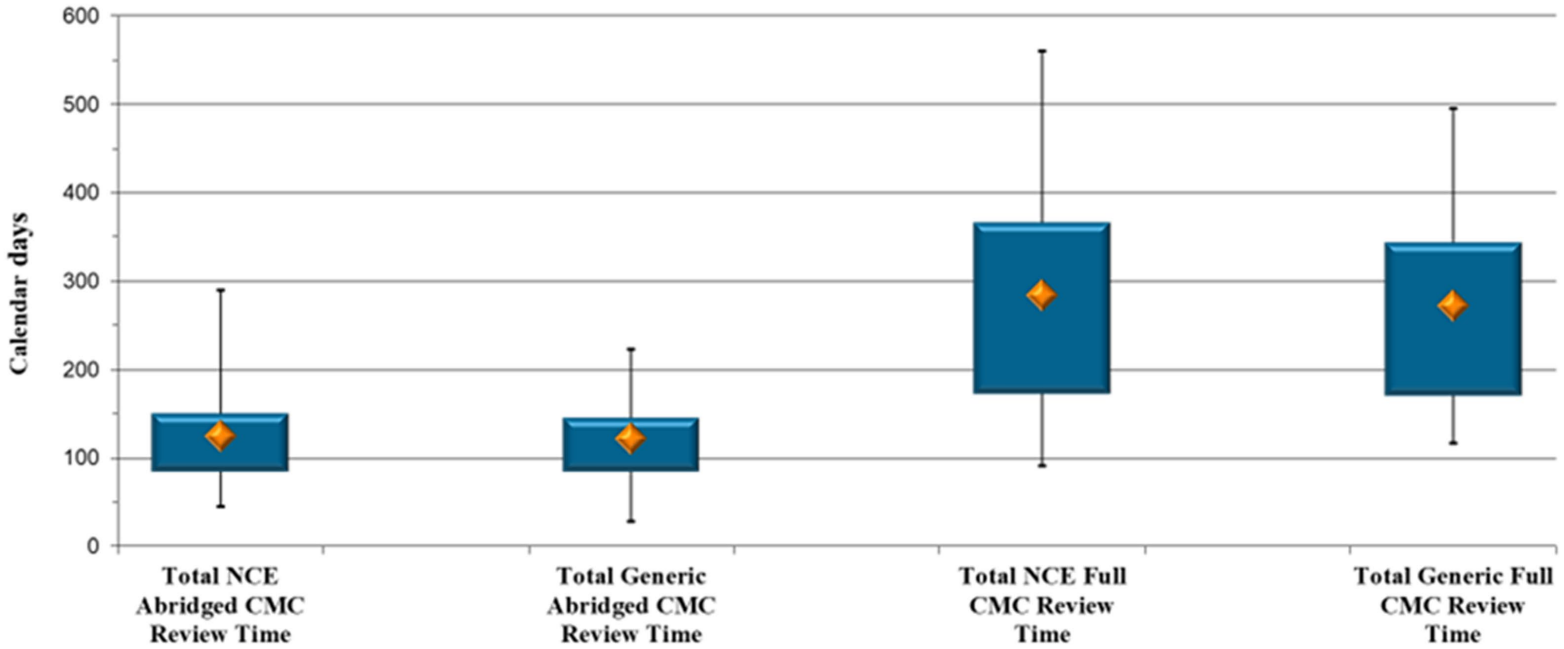
Comparison between the abridged and full Chemistry, Manufacturing and Controls evaluation timelines for New Chemical Entity and generic product applications, respectively.

A final analysis was performed to assess the comparability between timelines for abridged CMC review for NCEs and generic molecules vs. a full review for the two types of products (Figure 4).

The results suggest a comparability between the time taken for abridged CMC review for NCE and for generic molecule applications, namely a median of 125 vs. 122 calendar days, respectively. It is important to again note that SAHPRA reviews the CMC and BE data for generic product applications together and that reliance was also placed on previous assessment of BE studies by RRAs. A comparison of the full CMC review pathway yielded the same conclusion, in that full review timelines for the CMC data do not fluctuate excessively between the two types of molecules (a median of 284 calendar days for NCEs compared with 272 days for generic molecules), this despite the added BE component to be reviewed for generic product applications.

### 3.7. Assessor feedback on reliance review

Completed surveys were received from BCP CMC and clinical evaluators. The CMC assessor cohort comprised of internal SAHPRA, external domestic and international evaluators and these reviewers participated in the virtual focus group discussion. Further completed surveys regarding abridged clinical review were received from external SAHPRA evaluators.

## 4. Discussion

As part of the SAHPRA adoption of the WHO Global Benchmarking Tool (GBT) (18), the agency has current targeted timelines for new application reviews, measured as part of its key performance indicators (KPIs) (19). For NCEs, 80% of applications are to be finalized in 490 working days (equating to approximately 686 calendar days) (19). Even though the BCP full review of NCEs took a median of 619 calendar days, which is within target, it is important to note that the BCP was a sufficiently resourced project with a finite number of applications. The results from this study point to a 466-calendar day timeframe for authorization of an NCE via an abridged review route, which is significantly shorter than that by the MCC in 2017 (that is, 1,141 days) (14) and substantially faster than the SAHPRA current targeted timeframe for NCE review, compared with other countries in the region (20).

In terms of NCE applications, both SAHPRA CMC and clinical abridged reviews were shorter in duration than those of their fully reviewed counterparts. There was a time-reduction associated with abridged review across all measured timeframes, whether it was time spent by SAHPRA assessing the data, applicants providing responses to questions posed by SAHPRA, or CMC/clinical approvals and product authorization.

The results showed that the implementation of an abridged review was less successful for clinical assessment, and this was linked to the additional review step for clinical data for NCEs. All clinical decisions by reviewers are also tabled for endorsement to an Advisory Clinical Committee (ACC), which only meets on an *ad hoc* basis. This is coupled with a reticence to adopt reliance practices within this particular forum, which is evidenced by the high level of misalignment of SAHPRA vs. RRA labeling for NCEs. SAHPRA clinical evaluators were more restrictive in their conditions for use for innovator products, often not aligning with the RRA-approved information. The main areas of misalignment pertained to more restrictive therapeutic indications, additional contraindications, as well as precautions added for pregnant and lactating women. This approach by the reviewers reflects their practice of taking a more conservative attitude to the benefit-risk evaluation of products. This despite the availability of assessment reports from the EMA, with whom SAHPRA first and foremost aligns itself in terms of clinical assessment (2). Perhaps the EMA needs to carry out an approach in which it not only explains the benefit-risk evaluation but also the labeling rationale. That is, there might be a greater alignment if the reasoning from the initial assessment was better understood. The non-alignment may also be indicative of the level of trust of the clinical evaluators in a specific RRA and/or in reliance practices *per se* or could be attributed to considerations pertaining to the therapeutic effect of a product in the South African-specific population. This area has, however, subsequently benefitted from a cultural transformation, which will hopefully bring about closer alignment with RRA labeling. However, despite these challenges, marketing authorization timelines were still significantly shorter for NCE applications that underwent both CMC and clinical abridged review compared with those fully reviewed.

Similar trends were observed when analyzing CMC and BE review timelines for generic product applications. Less than half the time was required to assess and approve CMC and BE data in generic product applications via an abridged review and applicants were able to provide responses to questions in a third of the time. The NCE and generic molecule data, furthermore, showed comparability regarding timeframes for abridged vs. full CMC review.

Please note that regarding the sample selection, the ideal would have been random selection in order to minimize selection bias; however, in this study that was not possible due the nature of the availability of the sample and the study requirement for the full and abridged review to be paired.

Reliance on the Pharmaceutical Inspection Convention and Pharmaceutical Inspection Co-operation Scheme (PIC/S) and RRAs when assessing the Good Manufacturing Practices (GMP) compliance of sites was found particularly effective in reducing marketing authorization timelines in the BCP. It takes SAHPRA approximately 6 months to finalize an inspection of a site where no reliance documentation is available. Furthermore, prior to the physical inspection of a site, there is a lag time in the facility being allocated an inspection slot due to limited resources. This can lead to long delays before an application is even allocated for CMC evaluation, which is dependent on the positive GMP status of the relevant facilities. In contrast, based on SAHPRA experience, the duration of a reliance-based review of the GMP compliance status of a site is generally between 2 and 3 h, thereby decreasing marketing authorization time of a product dramatically.

The survey and discussion group feedback from the BCP assessors corresponded with the above findings, in that they all highlighted the time-saving aspect of abridged review of NCE and generic registration applications. This was also enabled by the dedicated SAHPRA abridged review template (21), that guided them to selected critical areas to be assessed for data similarity. Overall, the level of information included in the RRA assessment reports was found to be sufficient to engender confidence that the dossier submitted to SAHPRA was the same as that authorized by the relevant RRA. However, the assessors pointed out the importance of applicants providing the full cycle of unredacted reports, as this was imperative for ensuring “sameness” of the product under review. Some evaluators also consulted PARs for supplementary information, which they found useful, and, from an abridged clinical review perspective, the EMA EPARs, as well as the European Union Heads of Medicines Agency Co-ordination Group for Mutual Recognition and Decentralized procedures – Human Mutual Recognition Information (EU HMA CMDh MRI) Product Index, were used as review tools, with the US FDA PARs also being consulted. The reviewers were of the opinion that the benefits gained from having access to the reference agency’s assessment report in turn supported quality decision-making practices.

The evaluators, furthermore, indicated that not all RRA reports were equally useful, with the level of redaction varying between agencies. One clinical evaluator remarked that the quality of the documentation submitted further depended on whether the applicant was “well known, well established, and well capitalized.” Applications received from multi-national pharmaceutical companies were more often supported with full sets of reports, justifications and the lists of queries raised by the RRA, together with the associated applicant responses.

A major concern among the assessors was the inclusion of variations in the applications to SAHPRA (especially variations in CMC), with these submitted to the RRA after the initial marketing authorization, whether already approved by or still under review with the RRA. The SAHPRA Reliance Guideline states that only RRA-approved variations may be included in an abridged review application, supported by the relevant approvals (17). However, this guideline was not always adhered to by applicants and it lengthened the abridged review, as approval documentation, if available, was time consuming to obtain. However, one assessor indicated that “…subsequent amendments to the product and declared differences should not be of any concern whatsoever, as [SAHPRA] has the expertise to responsibly evaluate the practically unavoidable subsequent variations. Subsequent product variations do not and should certainly not negate the worth of reliance review.”

It is, nevertheless, important that applicants thoroughly detail all additional variations included in an NRA product dossier, to allow assessors to determine similarity. Reviewers further reported on the value abridged assessment has for new assessors, as exposure to the RRA reports enhanced their knowledge of not only the assessment of new product applications, but also of good report writing and query formulation. Reliance review was touted as an excellent way to learn how other agencies review and authorize medicines.

Only 20–30% of the marketing authorization applications received in the BCP qualified for abridged review, where unredacted RRA assessment reports were obtained and submitted by the applicant. The BCP received more than 130 Letters of Access from applicants, allowing SAHPRA to directly contact RRAs, such as the EMA, the US FDA, the UK MHRA, Health Canada and the WHO, to obtain the relevant reports. However, fewer than 5 unredacted reports were shared with SAHPRA via direct engagement between the two parties. The lack of availability of RRA reports, especially for generic medicines, inhibits the implementation of reliance review in NRAs. This led to SAHPRA signing several Memoranda of Understanding with the RRAs with which it aligns, namely the US FDA, the EMA, the MHRA, the TGA and Swissmedic, among others, with a view of increasing cooperation between SAHPRA and the RRAs. It is anticipated that this will assist in improving access to RRA assessment reports.

Reflecting on the outcome of the Backlog Clearance Project presented earlier and taking into account the assessors’ feedback, in order to keep the momentum of the success, the following recommendations should be considered:

- **Cultural transformation** – Ensure that reviewers have subscribed to the concept of reliance, especially with regard to clinical assessment, as this necessitates a change in their mindset
- **Risk-based review** – Create a priority evaluation process, differentiating medicine applications to the model of review, which in turn will lead to operational efficiency
- **Reference agency assessment report** – Agencies implementing reliance should engage with the WHO-listed authorities by developing Memoranda of Understanding (MoUs) in order to gain access to unredacted assessment reports
- **Information management system** – It should be incumbent upon the agencies to establish robust electronic tracking systems in order to measure and monitor regulatory performance which in turn would underpin the success of reliance initiatives

## 5. Limitations of the study

In the current regulatory environment in Africa, establishment of reliance as a standard pathway in the review process meets with resistance due to a certain mindset. Therefore, in this study only those assessors who were willing to be engaged in the backlog project were involved. This could be considered as a limitation. Furthermore, at times there was a challenge to obtain unredacted assessment reports from reference agencies. In addition, where public assessment reports were taken into consideration, these often lacked the required details regarding the decision-making process.

## 6. Conclusion

With many medicine regulators struggling with application backlogs, National Regulatory Authorities are increasingly looking toward reliance and risk-based assessments to reduce these backlogs and expedite patients’ access to medicines. The conclusions drawn from this study confirm the resource-saving benefit of implementing reliance-based review practices, not just within SAHPRA, but in other low-to-middle income countries, with commensurate increased access of medicines to patients. This outcome is echoed in a previous internal study on reliance implementation within SAHPRA, which established that reliance translated into 68% quicker authorization times (22). As one of the levers to preventing and clearing medicine authorization backlogs, it is thus critical that reliance on prior work carried out by RRAs is implemented by national regulators. As the Kenyan Pharmacy & Poisons Board (PPB) candidly stated: “backlog is not just an administrative challenge but represents a public health crisis” (10). The World Health Organization emphasizes that “reliance is not a lesser form of regulatory oversight but rather a strategy for making the best use of the available resources in any setting” (3). Reliance practices have changed the regulatory landscape and practical implementation of facilitated review pathways has confirmed its ability to enhance health access across the globe.

## Data availability statement

The raw data supporting the conclusions of this article will be made available by the authors, without undue reservation.

## Author contributions

LD: Conceptualization, Data curation, Formal analysis, Writing – original draft, Writing – review & editing. BS-M: Conceptualization, Writing – review & editing. KO: Formal analysis, Writing – review & editing. YP: Data curation, Writing – review & editing. SS: Conceptualization, Formal analysis, Writing – original draft, Writing – review & editing. SW: Conceptualization, Formal analysis, Writing – original draft, Writing – review & editing.
